# An analysis of perioperative hidden blood loss in femoral intertrochanteric fractures: bone density is an important influencing factor

**DOI:** 10.1186/s12891-020-03922-x

**Published:** 2021-01-04

**Authors:** Haidong Cui, Kai Chen, Shujun Lv, Chaoqun Yuan, Youhua Wang

**Affiliations:** 1 Department of Orthopaedic Surgery, Hai’an People’s Hospital, Zhongba Road 17, Hai’an, 226600 Jiangsu China; 2 Department of Orthopaedic Surgery, Affiliated Hospital of Nantong University, Xisi Road 20, Nantong, 226001 Jiangsu China

**Keywords:** Intertrochanteric fracture, Hidden blood loss, Influencing factors, Bone density

## Abstract

**Background:**

To explore the influencing factors of perioperative hidden blood loss in intertrochanteric fractures.

**Method:**

We undertook a retrospective analysis from January 2016 to October 2019. Clinical data of 118 patients with intertrochanteric fractures were included. Hidden blood loss was calculated from the haematocrit changes before and after surgery using the Gross equation based on height, weight, and haematocrit (HCT) changes before and after surgery. Patients’ gender, age, presence of underlying diseases, fracture types, anaesthesia methods, time from injury to surgery, administration of antiplatelet drugs within 6 months before surgery, use of anticoagulant drugs after surgery, and bone density were statistically analysed. Factors having an effect on hidden blood loss were screened out. Then, hidden blood loss was used as the dependent variable, and each influencing factor was used in turn as the independent variable. Multivariate linear regression analysis was employed to analyse the related risk factors that affect hidden blood loss during the perioperative period of patients with intertrochanteric fractures.

**Result:**

The apparent blood loss during the operation was 203.81 ±105. 51 ml, and the hidden blood loss was 517.55±191.47 ml. There were significant differences in the hidden blood loss of patients with different fracture types (stable vs unstable), anaesthesia methods (general anaesthesia vs intraspinal anaesthesia), antiplatelet or postoperative anticoagulant drugs, and bone densities (*P*< 0.05). 05). Multiple linear regression analysis showed that internal fixation, age, fracture type, anaesthesia method, anticoagulant application, and bone density were related risk factors that affected hidden blood loss during the surgical treatment of intertrochanteric fractures.

**Conclusion:**

Hidden blood loss is the main cause of perioperative blood loss in intertrochanteric fractures, and the risk factors for hidden blood loss include internal fixation, fracture type (e.g., unstable), anaesthesia (e.g., intraspinal), and use of anticoagulant drugs. Specifically, we found that low bone density was a risk factor for hidden blood loss. It is not reliable to use apparent blood loss as the basis for fluid replacement and transfusion. We must fully consider the existence of hidden blood loss and intervene as soon as possible to prevent complications.

**Level of evidence:**

III

## Background

As the population ages, the incidence of femoral intertrochanteric fracture is increasing. Indeed, intertrochanteric fracture has become the most prevalent type of hip fracture among elderly individuals [1]. At present, surgery is the most common treatment for femoral intertrochanteric fracture [2]. During surgery, loss of blood is inevitable. In the past, dominant blood loss during operations has received great attention in clinical practice. However, the existence of perioperative hidden blood loss has been neglected frequently. However, the haemoglobin level of patients after surgery has a great influence on hidden blood loss [3]. At present, an increasing number of studies have focused on the influencing factors of hidden blood loss perioperatively. However, most of these reports are not comprehensive, especially with respect to the relationship between bone density and hidden blood loss. Therefore, we carried out this retrospective study to explore the influencing factors of hidden blood loss perioperatively and thereby provide a reference for clinical treatment.

## Methods

### General information

From January 2016 to October 2019, 118 patients with intertrochanteric fractures treated at Hai’an People’s Hospital were included. The inclusion criteria were as follows: (1) fresh intertrochanteric fractures without multiple fractures or pathological fractures; (2) no previous blood disease history and normal coagulative function on the preoperative test; and (3) routine blood examination performed preoperatively and on days 2 and 3 postoperatively. A total of 118 patients, 57 males and 61 females, met the inclusion criteria. The age distribution of patients was as follows: 31 patients < 60 years old and 87 patients ≥60 years old. There were 58 stable fractures (Evans type I, II) and 60 unstable fractures (Evans type III, IV) [4]. Anaesthesia method: Forty-seven patients received general anaesthesia, and 71 patients received intraspinal anaesthesia. Sixty-four patients were treated with anticoagulant drugs. There were 57 patients with hypertension and 60 patients with diabetes. All patients underwent dual energy X-ray absorptiometry to test their bone density before surgery. During the bone density subgroup analysis, the included patients were divided into three groups according to the criteria recommended by the WHO for the diagnosis of osteoporosis: the group with normal bone density (T-value > − 1.0), the osteopenia group (− 2.5< T-value < − 1.0), and the osteoporosis group (T-value < − 2.5).

### Detection index

Hidden blood loss = total blood loss - apparent blood loss + transfusion. Patient blood volume (PBV) =K1× height (h)^3^ +K2× weight (kg) +K3. For men, K1, K2 and K3 were 0.3669, 0.03219 and 0.6041, respectively. For women, K1, K2 and K3 were 0.3561, 0.03308, and 0.1833, respectively [5]. Total red blood cell (RBC) loss = preoperative blood volume (PBV) × (preoperative HCT- postoperative HCT). Total theoretical blood loss = total RBC loss/preoperative HCT. Actual perioperative blood loss = hidden blood loss + apparent blood loss. Apparent blood loss = intraoperative blood loss + volume of drainage. For patients requiring blood transfusions, 1 μl of concentrated RBC suspension is equivalent to 200 ml of standard RBC volume.

### Statistical analysis methods

SPSS 13.0 software was used for analysis. A statistical analysis was carried out on the variables of patient gender, age, weight, bone density, underlying diseases (hypertension, diabetes), fracture type, internal fixation method, anaesthesia method, use of anticoagulant medication, etc. The risk factors were analysed by multiple linear regression analysis, with hidden blood loss as the dependent variable and influencing factors as the independent variables. *P* < 0.05 was considered statistically significant.

## Results

### Blood loss

The intraoperative apparent blood loss was 203.81 ± 105.51 ml, while the hidden blood loss was 517.55 ± 191.47 ml.

### Comparison of hidden blood loss under different factors

The perioperative hidden blood loss of patients with femoral intertrochanteric unstable fractures was significantly greater than that of patients with stable fractures (*P* < 0.05). The hidden blood loss of patients using general anaesthesia was significantly greater than that of patients with spinal canal anaesthesia (*P* < 0.05). The hidden blood loss of patients using anticoagulant drugs was also significantly greater than that of the non-users (*P* < 0. 05). The hidden blood loss of patients equal to or older than 60 years old was significantly greater than that of patients younger than 60 years (*P* < 0.05). Specifically, patients with osteoporosis had significantly greater hidden blood loss than patients with normal bone density and osteopenia (*P* < 0.05) (Table 1).

**Table 1:** Comparison of hidden blood loss under different factors (X±s)

Variable	Number of cases	Hidden blood loss	t/F	P
Gender				
Male	57	529.28±193.17		
Female	61	506.48±190.79	0.648	0.518
Age (years)				
≥60	87	531.49±188.37		
<60	31	413.98±188.98	2.190	0.030
Time of the operation				
<3 h	78	511.37±188.37		
	40	529.58±200.87	−0.487	0.627
Fracture type				
Stable	58	437.97±164.04		
Unstable	60	536.84±163.26	−3.059	0.003
Anaesthesia				
General anaesthesia	47	596.20±177.26		
Intraspinal anaesthesia	71	465.48±183.64	−3.838	0.000
Hypertension				
Yes	60	530.90±194.70		
No	58	503.74±188.75	0.769	0.443
Diabetes				
Yes	57	527.35±202.12		
No	61	508.39±182.16	0.536	0.593
Bone mineral density				
Normal	23	391.43±145.44		
Osteopenia	46	497.42±181.97		
Osteoporosis	49	595.63±185.44	10.895	0.000
Use of anticoagulants				
Yes	64	559.17±190.16		
No	54	468.22±182.68	−2.635	0.010
BMI				
≥28	46	500.39±178.38		
<28	72	528.51±199.83	−0.777	0.439

### Analysis of risk factors affecting hidden blood loss

Multiple linear regression analysis was conducted with hidden blood loss as the dependent variable and influencing factors as the independent variables. The results showed that fracture type, anaesthesia mode, use of anticoagulant drugs, age and bone mineral density affected the perioperative hidden blood loss of patients with intertrochanteric fractures (*P* < 0.05) (Table 2).

**Table 2:** Analysis of the risk factors affecting hidden blood loss

Affecting Factors	Unstandardized Coefficients	Standard Error	Standardized Coefficients	t	P
Gender	0.029	0.021	0.027	1.380	0.170
Age	0.044	0.015	0.058	1.958	0.034
Time of the operation	−0.476	0.501	- 0.059	−0.951	0.344
Fracture type	1.794	0.194	0. 427	9.242	0.000
Anaesthesia	0.633	0.024	0. 493	25.938	0.000
Hypertension	0.002	0.220	0.000	0.010	0.992
Diabetes	0.023	0.055	0.014	0.427	0.670
Bone mineral density	−0.622	0.032	−0.482	−19.537	0.000
Use of anticoagulants	−0.014	0.004	−0.059	−3.212	0.002
BMI	0.001	0.010	0.002	0.099	0.922

## Discussion

Femur intertrochanteric fractures have a high incidence in the elderly population, and most of these fractures are comminuted. Displacement is a factor negatively affecting the outcome of intertrochanteric fractures [6]. Currently, surgery is the preferred treatment method for intertrochanteric fractures, with satisfactory curative effects and low complication rates [6]. There are mainly two kinds of surgical approaches: extramedullary fixation and intramedullary fixation [7]. Intramedullary fixation is becoming the preferred method for the surgical treatment of intertrochanteric fractures. Regarding the outcomes and complications of femoral intertrochanteric fractures, the fracture type, blood loss, operation, level of irisin hormone and presence of a pseudoaneurysm are important influencing factors [7, 8].

Perioperative blood loss may lead to many complications and a poor prognosis [9]. Such loss also increases the incidence of infection and deep vein thrombosis. Moreover, the patient’s mortality rate rises [9]. Therefore, it is important to identify the causes of perioperative blood loss. Hidden blood loss accounts for a high percentage of the total perioperative blood loss in patients with intertrochanteric fractures. If the presence of hidden blood loss is not considered, it will often lead to anaemia or low blood volume in patients. This will affect postoperative recovery and even cause serious consequences. At present, the biological mechanism of hidden blood loss has not been clearly studied. The existing studies report that the causes of hidden blood loss include the following aspects: 1) blood that enters the tissue or the joint cavity and thus no longer participates in the humoral circulation [10] and 2) RBC haemolysis caused by injury. Some stressful events that occur during the operation, such as trauma and anaesthesia, may lead to changes in the internal blood environment and subsequent RBC peroxidation damage. On the other hand, RBC damage during the process of autologous blood transfusion and other factors may cause haemolysis, thus making hidden blood loss more serious. 3) Gastrointestinal stress ulcers caused by trauma and surgery will also cause hidden blood loss.

In this study, factors affecting perioperative hidden blood loss in patients with intertrochanteric fractures were analysed. We found that unstable fractures, advanced age, osteoporosis and general anaesthesia were independent risk factors for increasing hidden blood loss. Kumar et al. observed significant differences in the amount of hidden blood loss in patients with different fracture types [11]. Some researchers found that the hidden blood loss in patients with Evans I and II type fractures was significantly lower than that in patients with III and IV type fractures. They also found that there was a relationship between mean platelet volume and reoperation occurrence [4]. The results of our study showed that the hidden blood loss in patients with unstable fractures was significantly higher than that in patients with stable fractures, which confirms present research. It seems that there is a certain correlation between fracture type and hidden blood loss [12–15]. Therefore, we should pay attention to reviewing patients’ routine blood tests and take timely blood transfusions during treatment for complicated fracture types.

In our study, we found that the selection of anaesthesia, the use of anticoagulant drugs and age are also key factors affecting perioperative hidden blood loss. This may be related to the fact that the antifibrinolytic ability of patients under general anaesthesia is lower than that of patients under epidural anaesthesia [16]. During the treatment of lower limb surgery patients, a certain amount of anticoagulant medication will be used to prevent the formation of venous thrombosis. Thus, the amount of hidden blood loss will also increase. In terms of age, in patients with total hip replacement, researchers found that the amount of hidden blood loss in patients older than 70 years was significantly higher than that in patients younger than 70 years [17]. In our study, the amount of hidden blood loss was significantly higher in patients over 60 years old than in patients under 60 years old. The effect of gender on the amount of hidden blood loss is still controversial. Most researchers believe that there is no significant difference between male and female patients with intertrochanteric femur fracture [18]. The results of our study indicate that gender is not a risk factor for hidden blood loss, which is consistent with most reports. In addition, some researchers believe that there are significant differences in the amount of hidden blood loss between different internal fixators, such as proximal femoral nail antirotation and the dynamic hip screw [19, 20]. Some investigators also undertook studies regarding the time of occurrence of hidden blood loss. They found that hidden blood loss occurred just after injury and ended on postoperative day 2 [21, 22].

At present, there are few reports on the effect of bone density on perioperative blood loss in patients with intertrochanteric fractures. In our study, we found significant differences in the amount of hidden blood loss between groups with different bone mineral densities. This may be because, in patients with low bone density, the bone trabeculae become thin, and some even fracture. Osteoporosis may cause enlargement of the bone marrow space; the appearance of micropores and cancellation of the bone cortex; and the enlargement of periosteal pores, cortical pores, and endosteum pores. As a result of the above changes, osteoporosis maight increase the amount of blood loss during the perioperative period. In addition, patients with osteoporosis are generally older, their body’s self-regulation ability is weakened, and their vascular elasticity is poor, which can also increase the risk of hidden blood loss. We should assess the bone density of each patient before surgery according to the imaging examination and the patient’s medical history.

The advantages of this study are that bone density is an important influencing factor. This might affect the clinical strategy for intertrochanteric bone fracture. For example, for osteoporosis patients, we should improve their bone density to avoid more hidden blood loss. The limitations of this study are as follows: the time span of each patient was different, and the standards for some influencing factors might be different. Other potential factors, such as American Society of Anesthesiologists (ASA) grade and other complications, were not included in the study. The cases were obtained from multiple doctors, and there might have been differences during surgery.

## Conclusion

Hidden blood loss is the main cause of perioperative blood loss in intertrochanteric fractures. The risk factors for hidden blood loss include internal fixation, fracture type, anaesthesia, and use of anticoagulant drugs. Specifically, we found that bone density was a risk factor for hidden blood loss. It is not reliable to use apparent blood loss as the basis for fluid replacement and transfusion. We must fully consider the existence of hidden blood loss and intervene as soon as possible to prevent complications.

## Data Availability

Author Haidong Cui can provide original data upon reasonable request. His email is 2036126481@qq.com.

## Abbreviations

HCT: Haematocrit
WHO: World Health Organization
PVB: Patient blood volume
RBC: Total red blood cell
BMI: Body Mass Index
